# Exposure to and experiences of violence among adolescents in lower socio-economic groups in Johannesburg, South Africa

**DOI:** 10.1186/s12889-015-1780-8

**Published:** 2015-05-01

**Authors:** Kennedy N Otwombe, Janan Dietrich, Kathleen J Sikkema, Jenny Coetzee, Kathryn L Hopkins, Fatima Laher, Glenda E Gray

**Affiliations:** Perinatal HIV Research Unit, Faculty of Health Sciences, University of the Witwatersrand, P.O Box 114, , Diepkloof, 1864 Johannesburg, South Africa; Department of Psychology and Neuroscience, Duke Global Health Institute, Duke University, Durham, North Carolina USA

**Keywords:** Adolescents, Low socio-economic settings, Witnessing violence, Experiencing violence

## Abstract

**Background:**

We explored exposure to and experiences of violence and their risk factors amongst ethnically diverse adolescents from lower socio economic groups in Johannesburg.

**Methods:**

This cross-sectional study recruited a stratified sample of 16–18 year old adolescents from four low socio-economic suburbs in Johannesburg to reflect ethnic group clustering. We collected socio-demographic, sexual behaviour, alcohol and drug use and trauma events data. Proportions and risk factors were assessed by chi-square and logistic regression.

**Results:**

Of 822 adolescents, 57% (n = 469) were female. Approximately 62% (n = 506) were Black, 13% (n = 107) Coloured, 13% (n = 106) Indian and 13% (n = 103) White. Approximately 67% (n = 552) witnessed violence to a non-family member, 28% (n = 228) experienced violence by a non-family member, and 10% (n = 83) reported sexual abuse. Multivariate analysis determined that witnessing violence in the community was associated with being Black (OR: 4.6, 95%CI: 2.7-7.9), Coloured (OR: 3.9, 95%CI: 2.0-7.4) or White (OR: 8.0, 95%CI:4.0-16.2), repeating a grade (OR: 1.5, 95%CI: 1.01-2.1), having more than one sexual partner (OR: 1.7, 95%CI: 1.1-2.5) and ever taking alcohol (OR: 2.1, 95%CI: 1.5-2.9). Witnessing violence in the family was associated with being female (OR: 1.8, 95%CI: 1.3-2.6), being Black (OR: 2.2, 95%CI: 1.1-4.1), or White (OR: 3.0, 95%CI: 1.4-6.4), repeating a grade (OR: 1.6, 95%CI: 1.1-2.2) and ever taking alcohol (OR: 2.9, 95%CI: 2.0-4.3).

**Conclusions:**

In low socio-economic areas in Johannesburg, Black, White and Coloured adolescents experience a high burden of violence. Interventions to mitigate the effects of violence are urgently required.

## Background

Violence is defined as the intentional use of physical force against another person within the family or community that may result in injury or psychological harm [1,2]. Community violence is defined as witnessing or experiencing violence by individuals unrelated to the victim, while family violence is witnessing or experiencing violence in the family. The extent of violence among those aged between 10 and 29 years is well described [1,2], and the World Health Organisation listed interpersonal violence as the fifth leading cause of death amongst adolescents in 2012 [3]. Because of its contribution to mortality, morbidity and long-term health implications, violence is now widely recognized as a public health priority, especially in adolescents [1,4-8].

Although violence cuts across all socio-economic groups, it is more prevalent in lower socio-economic settings [5]. Based on South African data from the year 2000 in 15–29 year olds, homicide or interpersonal violence among males was nine times higher than the global average and five times higher in females [9]. In another South African study with 12 to 17 year olds, those from a lower socio-economic group had an increased odds of experiencing violence [10]. In lower socioeconomic groups in Cape Town, 48% of children aged 8–13 years have witnessed a murder, while 94% of 14–21 year olds have seen somebody being beaten in their neighbourhood [11,12].

The association between gender and violence is established [13,14]. In a dating violence study among adolescents aged 10–18 years in South Africa and Tanzania, males were associated with perpetration and females with victimization [15]. On further sub-group analysis focussing on the Cape Town adolescents, perpetration of violence was associated with females [15]. In another study covering six sub-Saharan Africa countries (Liberia, Ghana, Nigeria, Uganda, Zambia and Zimbabwe), there were mixed findings on the association between gender and sexual abuse [16]. Adolescents may use violence to show power and control while promoting status and self-image [10,17].

The role of ethnicity as a risk factor for violence in South African adolescents remains unclear. In South Africa, where apartheid laws once separated ethnic groups, communities still largely live within their ethnic groups. A South African study with school-going adolescents between 15 and 18 years of age from two ethnic groups found no association between ethnicity and violence [18]. In the US, school-going Black/African American and Latino American adolescents have a higher odds of experiencing violence when associating with White American adolescents [19].

Violence may be fuelled by use of substances such as alcohol and drugs due to experimentation by adolescents. Alcohol consumption in South Africa in ≥ 15 year olds is classified as among the highest in the world [17,20] with high homicide levels related to alcohol [20,21]. Due to their developmental stage, adolescents commonly engage in excessive drinking that may lead to aggressive and violent behaviour [5,22-24]. In various cultural contexts, including South Africa and the U.S., substance use is associated with violence [25-27].

Gender based violence is exacerbated by risky sexual behaviour such as multiple sexual partnerships that could lead to negative health outcomes [28]. The association between violence and risky sexual behaviour such as multiple sexual partnerships in adolescents is well documented. Female adolescents have previously reported violent behaviour in males when questioned about their fidelity [4,29,30].

Little cross-cultural research on the epidemiology of adolescent violence in South Africa from low socio-economic settings has been reported. Furthermore no study on adolescent violence has reported on the four main ethnic groups, as defined by South Africa’s historical past: Blacks, Coloureds (a South African legal term for individuals of mixed race), Indians and Whites. Prior research on violence from South Africa has focussed on Blacks or Coloureds or both with limited information on Indians and Whites from low socio-economic settings. In addition, most previous studies have been conducted in Cape Town and there is much less data from Johannesburg, although it is a far larger urban centre. We hypothesise that in South Africa, ethnicity is important because of the differential access to opportunities [31,32] such as education and healthcare due to the ethnic-related structuring of communities in which people live. These opportunities affect exposure to violence and access to healthcare in the event of an injury. This study explores exposure to and experiences of violence and their risk factors amongst adolescents from different ethnic groups in low-socioeconomic settings of Johannesburg, South Africa.

## Methods

### Participants

This cross-sectional study recruited a stratified sample of adolescents between 16 and 18 years of age from October 2008-October 2009 from four pre-identified low socio-economic suburbs of Johannesburg: Soweto, Eldorado Park, Lenasia, and Brixton. Black adolescents were recruited from each of the forty townships comprising Soweto, which were considered a stratum. Coloured, Indian, and White adolescents from Eldorado Park, Lenasia, and Brixton respectively, were stratified by area.

### Study setting

#### Soweto

Approximately 1.3 million people live in Soweto, Johannesburg where there is a spectrum of formal and informal settlements. Between 600 000 to 1 million residents are regarded to live in poverty and the average household size is 4.2 square meters. The population is predominantly Black.

#### Lenasia

Lenasia is a formerly Indian neighbourhood south of Soweto with an estimated population of 90 000 people living in informal and formal settlements.

#### Eldorado Park

Eldorado Park is an area with a population, that is predominantly of the Coloured race, of approximately 350 000 people living in formal low-cost housing. It is surrounded by informal settlements.

#### Brixton

An estimated 4067 people live in Brixton, an area with 1262 formal households and the population is predominantly White. Brixton also has communal houses occupied by university students.

#### Procedures

Participants were recruited through a stratified sampling approach within the targeted neighbourhoods. This analysis was part of a larger study that investigated risk factors for HIV among adolescents to develop an adapted risk reduction counselling tool for adolescents [33,34]. In the primary study, enrolment was done using a 60:40% split because females are disproportionately affected by HIV in South Africa. Approximately 1184 adolescents were approached but 362 were not enrolled for various reasons: not interested (n = 157), expressed interest but failed to show up for their scheduled interview (n = 203) and unknown (n = 2). A minimum of twelve adolescents were purposively recruited from the 40 stratum in Soweto and approximately 100 per area were enrolled from Eldorado Park, Lenasia and Brixton. Purposive sampling was adopted to allow enrolment of those referred into the study by their friends or school mates. At recruitment, participants were informed that the study was about understanding young people (adolescents), their relationships, sexual behaviour, substance use and assessing their psychosocial situations. Recruitment strategies included fieldworkers targeting areas near high schools, youth organizations, malls, and shops, with follow up via telephone to schedule interviews. Interviews were conducted in English, although fieldworkers were fluent in relevant local languages in case further elaboration was needed.

Participant age was verified with identity or birth certificate documents. Participants completed interviewer-administered questionnaires that took approximately 90 minutes to complete. Interviews were conducted at a private venue, either a designated location near the participant’s home or at the Perinatal HIV Research Unit (PHRU), at Chris Hani Baragwanath Academic Hospital in Soweto. Participants were reimbursed ZAR50 (~USD 7).

Written consent was obtained from each participant for their information to be stored in the PHRU database and used for research purposes. Those younger than 18 years required written parental consent with participant assent. Ethical approval for consent and assent was obtained from institutional review boards of the University of the Witwatersrand and Duke University.

### Measures

#### Socio-demographic information

Data was collected on gender, age, ethnic group, schooling history and source of water. Adolescents were also asked whether their parents were alive, parent/guardian education level, marital status and head of household.

#### Sexual behavior

Participants reported whether they ever had vaginal and/or anal intercourse; with sexually active defined as one or both. Number of lifetime sexual intercourse partners was assessed and reported as none or one (assumed to be low-risk) versus more than one.

#### Alcohol and drug use

Alcohol and drug use were assessed by the items: “Ever had alcohol” and “had alcohol in the past six months” and “Ever had drugs” and “had drugs in the past six months”. A positive response of “Yes” was required to be classified in the affirmative. Each item was assessed individually.

#### Experience of traumatic events

A modified and shortened version of the Traumatic Events Questionnaire that has previously been used in South Africa [35], was administered that assessed exposure and experience of traumatic events in the community and at home. In the modified version, the items assessed were; “ever seen or witnessed acts of violence in the community” and “ever seen or witnessed acts of violence in the family”.

### Statistical analysis

Median and interquartile ranges were used to describe age while frequencies and associated percentages were determined for categorical variables to describe participant characteristics. Participant characteristics were described by socio-demographic variables such as gender, ethnicity, education such as repeating a grade at school and parents level of education, whether parents were alive and their marital status and stating the head of household. Socio-economic status was assessed by the variable source of water. Behavioural variables included number of sexual partners, alcohol and drug use. The comparison of responses to the violence items by gender was done using the chi-square test of proportions. Univariate and multivariate logistic regressions were used to determine factors associated with violence. At the univariate level, the following variables were used: Gender, ethnicity, repeated a grade, parents alive, ever had alcohol and had alcohol in the past 6 months, ever used drugs, number of sexual partners and sexually active. Four regression models were fitted for risk factors of violence; Ever seen an act of violence in the community, Ever experienced an act of violence in the community, Ever seen violence in the family and Ever experienced violence in the family. Due to space limitations, only multivariate results for each are presented. The stepwise selection procedure was used to select variables for inclusion into the multivariate model while the Hosmer-Lemeshow statistic was used for model diagnostics. Statistical analysis was two-sided and performed at a 5% level of significance using SAS Enterprise Guide version 5.1 (Statistical Analysis Software Institute, Cary, NC, USA).

## Results

### Demographics and sexual behaviour

**Figure 1:**
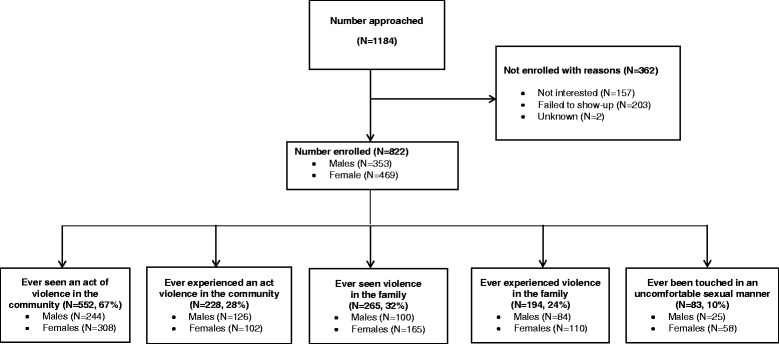
Participant Disposition Flow Chart of Violent Experiences.

Of 1184 adolescents approached, 69% (n = 822) were enrolled; 43% (n = 353) males and 57% (n = 469) females (Figure 1). Approximately 62% (n = 506) were Black, 13% (n = 107) Coloured, 13% (n = 106) Indian and 13% (n = 103) White. The overall median (IQR) age was 17 (16–18) years and 67% (n = 551) had never repeated a grade at school. About 42% (n = 348) had married parents and 54% (n = 447) were from female-headed households. Most adolescents (53%, n = 436) had parents with secondary school education.

### Exposure to and experience of violence

Approximately 67% (n = 552) reported witnessing an act of violence to a non-family member while 28% (n = 228) experienced an act of violence by a non-family member (Figure 1). Seeing violence happen to another family member and experiencing violence by a family member was reported by 32% (n = 265) and 24% (n = 194) adolescents respectively. The distribution of various acts of violence is presented in Table 1. Acts of violence occurred at a median age of 14 and 15 years. Those who reported seeing an act of violence to a non-family member, another family member or by a family member were 54% (n = 290), 44% (n = 114) and 47% (n = 91) respectively. Among those reporting witnessing violence happening to another family member, 40% (n = 106) indicated witnessing parents involved in physical fighting. One in ten (n = 83) adolescents reported being touched sexually of which 70% (58/83) were females, 59% (49/83) were Black and 22% (18/83) were White. Among them, 71% (35/49) and 67% (12/18) were Black and White female adolescents respectively. Surprisingly, the proportion reporting sexual violence was higher amongst White adolescents (17%, 18/103). Of those reporting being touched in a sexual way that made them uncomfortable, 69% (57/83) were coerced to promise never to tell anyone about their experience or were threatened and 59% (49/83) reported telling someone about their experience.

**Table 1:** **Traumatic experiences (Total = 822)**

	**Ever seen an act of violence in the community (n = 552, 67%)**	**Ever experienced an act of violence in the community (n = 228, 28%)**	**Ever seen violence in the family (n = 265, 32%)**	**Ever experienced violence in the family (n = 194, 24%)**	**Ever been touched in an uncomfortable sexual way (n = 83, 10%)**
**Gender**					
Male (n = 353, 43%)	244 (69)	126 (36)	100 (28)	84 (24)	25 (7)
Female (n = 469, 57%)	308 (66)	102 (22)	165 (35)	110 (23)	58 (12)
**Ethnic group**					
Black (n = 506, 62%)	365 (72)	133 (26)	175 (35)	136 (27)	49 (10)
Coloured (n = 107, 13%)	71 (66)	27 (25)	35 (33)	20 (19)	5 (5)
Indian (n = 106, 13%)	36 (34)	23 (22)	17 (16)	13 (12)	11 (10)
White (n = 103, 13%)	80 (78)	45 (44)	38 (37)	25 (24)	18 (17)
**Repeated Grade**					
Never repeated (n = 551, 67%)	353 (64)	121 (22)	161 (29)	117 (21)	50 (9)
Repeated (n = 271, 33%)	199 (73)	107 (39)	104 (38)	77 (28)	33 (12)
**Parents Alive**					
Both parents alive (n = 548, 67%)	352 (64)	144 (26)	169 (31)	113 (21)	55 (10)
Single parent alive (n = 230, 28%)	169 (73)	67 (29)	79 (34)	66 (29)	22 (10)
None alive (n = 44, 5%)	31 (70)	17 (39)	17 (39)	15 (34)	6 (14)
**Parents Marital Status**					
Married (n = 348, 42%)	212 (61)	92 (26)	98 (28)	73 (21)	36 (10)
Single (n = 213, 26%)	161 (76)	57 (27)	71 (33)	56 (26)	21 (10)
Other (n = 164, 20%)	114 (70)	48 (29)	66 (40)	35 (21)	14 (9)
**Household Head**					
Female (n = 447, 54%)	317 (71)	134 (30)	155 (35)	121 (27)	49 (11)
Male (n = 373, 45%)	233 (62)	93 (25)	109 (29)	71 (19)	34 (9)
**Parent’s Education Level**					
Primary (n = 29, 4%)	12 (41)	7 (24)	11 (38)	7 (24)	2 (7)
Secondary (n = 149, 18%)	104 (70)	38 (26)	50 (34)	37 (25)	15 (10)
Matric (n = 287, 35%)	194 (68)	82 (29)	88 (31)	71 (25)	33 (11)
Post-School Training (n = 102, 12%)	62 (61)	25 (25)	25 (25)	18 (18)	6 (6)
Don’t know (n = 255, 31%)	180 (71)	76 (30)	91 (36)	61 (24)	27 (11)
**Source of water**					
Tap water in home (n = 805, 98%)	543 (67)	223 (28)	259 (32)	190 (24)	82 (10)
Community tap/Other (n = 11, 1%)	6 (55)	5 (45)	4 (36)	3 (27)	1 (9)
**Sexually active**					
Yes (n = 369, 45%)	273 (74)	127 (34)	137 (37)	95 (26)	46 (12)
No (n = 453, 55%)	279 (62)	101 (22)	128 (28)	99 (22)	37 (8)
**Number of sexual partners**					
None or one (n = 564, 69%)	356 (63)	136 (24)	171 (30)	122 (22)	52 (9)
More than one (n = 258, 31%)	196 (76)	92 (36)	94 (36)	72 (28)	31 (12)
**Ever had alcohol**					
Yes (n = 539, 66%)	404 (75)	186 (35)	213 (40)	148 (27)	57 (11)
No (n = 280, 34%)	148 (53)	42 (15)	52 (19)	46 (16)	25 (9)
**Had alcohol in the past 6 months**					
Yes (n = 430, 53%)	339 (79)	156 (36)	171 (40)	128 (30)	43 (10)
No (n = 388, 47%)	212 (55)	72 (19)	94 (25)	65 (17)	39 (10)
**Ever had drugs**					
Yes (n = 166, 20%)	127 (77)	73 (44)	69 (42)	47 (28)	22 (13)
No (n = 651, 80%)	423 (65)	153 (24)	196 (30)	146 (22)	59 (9)
**Had drugs in the past 6 months**					
Yes (n = 108, 13%)	78 (72)	45 (42)	40 (37)	30 (28)	15 (14)
No (n = 708, 87%)	472 (67)	182 (26)	224 (32)	164 (23)	66 (9)

### Alcohol and drugs

Of those ever taking alcohol, 71% (n = 359) were Black, 66% (n = 71) Coloured, 44% (n = 46) Indian and 62% (n = 64) White. Of those ever taking drugs, 17% (n = 86) were Black, 21% (n = 22) Coloured, 32% (n = 34) Indian and 25% (n = 25) White. Females reporting use of alcohol in the past 6 months were more likely to report seeing violence in the family (100 (48.8%) vs. 71 (31.7%); p = 0.0003).

### Violence/trauma by gender

Female adolescents with married parents were less likely to witness acts of violence in the community (56.5%, n = 109 vs. 66.9%, n = 103; p = 0.0482) while Black male adolescents were more likely to witness acts of violence in the community compared to females (33.7%, n = 70 vs. 21.4%, n = 63; p = 0.002). Males were more likely to witness acts of violence in the community, regardless of whether they repeated a grade (p = 0.0451) or had one (p = 0.0218)/both parents alive (p = 0.0002) (Table 2). Females repeating a grade (p = 0.004), with primary school educated parents (p = 0.0049), sexually active (p = 0.0318) or have none/one sexual partner (p = 0.0036) were more likely to witness violence in the family compared to males. Similarly those not repeating a grade (p = 0.2) and having parents with secondary school (p = 0.1) or higher education (p = 0.5) were less likely to experience violence in the family.

**Table 2:** **Violence/Trauma experience by gender**

**Variable**	**Ever seen any violence**			**Ever seen an act of violence in the community**			**Ever experienced an act violence in the community**			**Ever seen violence in the family**			**Ever experienced violence in the family**		
	**Male**	**Female**	**p-value**	**Male**	**Female**	**p-value**	**Male**	**Female**	**p-value**	**Male**	**Female**	**p-value**	**Male**	**Female**	**p-value**
	**N (%)**	**N (%)**		**N (%)**	**N (%)**		**N (%)**	**N (%)**		**N (%)**	**N (%)**		**N (%)**	**N (%)**	
**Ethnic group**															
Black	171 (82.2)	238 (79.9)	0.5	156 (75.0)	209 (70.4)	0.3	70 (33.7)	63 (21.4)	0.0021	64 (30.9)	111 (37.9)	0.1	58 (27.9)	78 (26.3)	0.7
Coloured	35 (77.8)	44 (71.0)	0.4	29 (64.4)	42 (67.7)	0.7	14 (31.1)	13 (21.0)	0.2	14 (31.1)	21 (33.9)	0.8	10 (22.2)	10 (16.1)	0.4
Indian	23 (48.9)	29 (49.2)	0.98	19 (40.4)	17 (29.3)	0.2	14 (29.8)	9 (15.3)	0.1	5 (10.6)	12 (21.1)	0.2	5 (10.6)	8 (13.6)	0.6
White	45 (84.9)	43 (86.0)	0.9	40 (75.5)	40 (80.0)	0.6	28 (52.8)	17 (34.0)	0.1	17 (32.7)	21 (43.8)	0.3	11 (20.8)	14 (28.0)	0.4
**Repeated Grade in School**															
Never repeated	148 (74.4)	250 (71.0)	0.4	134 (67.3)	219 (62.6)	0.3	57 (28.6)	64 (18.3)	0.0049	52 (26.3)	109 (31.6)	0.2	48 (24.1)	69 (19.6)	0.2
Repeated	126 (81.8)	104 (88.9)	0.1	110 (71.4)	89 (76.1)	0.4	69 (44.8)	38 (32.8)	0.0451	48 (31.4)	56 (48.7)	0.0040	36 (23.4)	41 (35.3)	0.0311
**Parents Alive**															
Both parents alive	179 (75.2)	221 (71.3)	0.3	160 (67.2)	192 (62.1)	0.2	82 (34.5)	62 (20.1)	0.0002	64 (27.1)	105 (34.2)	0.1	48 (20.2)	65 (21.0)	0.8
Single parent alive	78 (81.3)	112 (83.6)	0.6	71 (74.0)	98 (73.1)	0.9	36 (37.5)	31 (23.5)	0.0218	29 (30.2)	50 (39.1)	0.2	27 (28.1)	39 (29.3)	0.8
None alive	17 (89.5)	21 (84.0)	0.6	13 (68.4)	18 (75.0)	0.6	8 (42.1)	9 (36.0)	0.7	7 (36.8)	10 (40.0)	0.8	9 (47.4)	6 (24.0)	0.1
**Parents Marital Status**															
Married	112 (72.7)	131 (67.5)	0.3	103 (66.9)	109 (56.5)	0.0482	56 (36.4)	36 (18.7)	0.0002	39 (25.7)	59 (30.9)	0.3	32 (20.8)	41 (21.1)	0.9
Single	71 (88.8)	111 (83.5)	0.3	63 (78.8)	98 (73.7)	0.4	30 (37.5)	27 (20.5)	0.0067	24 (30.0)	47 (35.6)	0.4	18 (22.5)	38 (28.6)	0.3
Other	52 (76.5)	76 (79.2)	0.7	44 (64.7)	70 (73.7)	0.2	21 (30.9)	27 (28.1)	0.7	23 (33.8)	43 (45.7)	0.1	16 (23.5)	19 (19.8)	0.6
**Household Head**															
Female	149 (81.4)	211 (79.9)	0.7	130 (71.0)	187 (71.1)	0.98	70 (38.3)	64 (24.4)	0.0018	55 (30.1)	100 (38.8)	0.1	45 (24.6)	76 (28.9)	0.3
Male	124 (73.4)	142 (69.6)	0.4	113 (66.9)	120 (59.1)	0.1	56 (33.1)	37 (18.2)	0.0009	44 (26.3)	65 (32.3)	0.2	38 (22.5)	33 (16.2)	0.1
**Parents Educational Level**															
Primary School	85 (76.6)	138 (79.8)	0.5	73 (65.8)	119 (68.8)	0.6	41 (36.9)	42 (24.3)	0.0221	29 (26.4)	73 (42.9)	0.0049	19 (17.1)	49 (28.3)	0.0308
Up to Secondary School	156 (78.8)	177 (74.4)	0.3	143 (72.2)	155 (65.7)	0.1	70 (35.4)	50 (21.2)	0.0010	61 (31.0)	77 (32.9)	0.7	56 (28.3)	52 (21.9)	0.1
Post-school training	33 (75.0)	39 (67.2)	0.4	28 (63.6)	34 (58.6)	0.6	15 (34.1)	10 (17.5)	0.1	10 (22.7)	15 (26.8)	0.6	9 (20.5)	9 (15.5)	0.5
**Sexually Active**															
Yes	165 (82.9)	145 (85.3)	0.5	151 (75.9)	122 (72.2)	0.4	71 (35.7)	56 (32.9)	0.6	64 (32.3)	73 (43.2)	0.0318	46 (23.1)	49 (28.8)	0.2
No	109 (70.8)	209 (69.9)	0.8	93 (60.4)	186 (62.4)	0.7	55 (35.7)	46 (15.5)	<.0001	36 (23.5)	92 (31.6)	0.1	38 (24.7)	61 (20.5)	0.3
**Number of sexual partners**															
None or one	140 (72.5)	269 (72.5)	0.99	120 (62.2)	236 (63.8)	0.7	65 (33.7)	71 (19.3)	0.0002	44 (23.0)	127 (35.1)	0.0036	43 (22.3)	79 (21.4)	0.8
More than one	134 (83.8)	85 (86.7)	0.5	124 (77.5)	72 (74.2)	0.5	61 (38.1)	31 (31.6)	0.3	56 (35.0)	38 (38.8)	0.5	41 (25.6)	31 (31.6)	0.3
**Ever had alcohol**															
Yes	218 (82.3)	237 (86.2)	0.2	200 (75.5)	204 (74.5)	0.8	106 (40.0)	80 (29.2)	0.0084	84 (31.8)	129 (47.4)	0.0002	66 (24.9)	82 (29.8)	0.2
No	56 (63.6)	116 (60.1)	0.6	44 (50.0)	104 (54.2)	0.5	20 (22.7)	22 (11.5)	0.0150	16 (18.4)	36 (19.3)	0.9	18 (20.5)	28 (14.6)	0.2
**Had alcohol in the past 6 months**															
Yes	193 (86.2)	184 (88.9)	0.4	180 (80.4)	159 (77.2)	0.4	89 (39.7)	67 (32.5)	0.1	71 (31.7)	100 (48.8)	0.0003	59 (26.3)	69 (33.3)	0.1
No	81 (62.8)	168 (64.6)	0.7	64 (49.6)	148 (57.1)	0.2	37 (28.7)	35 (13.6)	0.0003	29 (22.8)	65 (25.7)	0.5	25 (19.4)	40 (15.4)	0.3
**Ever had drugs**															
Yes	90 (82.6)	53 (91.4)	0.1	83 (76.1)	44 (77.2)	0.9	50 (45.9)	23 (41.1)	0.6	41 (37.6)	28 (49.1)	0.2	27 (24.8)	20 (35.1)	0.2
No	184 (75.4)	298 (73.0)	0.5	161 (66.0)	262 (64.4)	0.7	76 (31.1)	77 (18.9)	0.0004	59 (24.4)	137 (34.3)	0.0085	57 (23.4)	89 (21.8)	0.6
**Had drugs in the past 6 months**															
Yes	64 (80.0)	26 (89.7)	0.2	58 (72.5)	20 (71.4)	0.9	33 (41.3)	12 (42.9)	0.9	25 (31.3)	15 (53.6)	0.0353	20 (25.0)	10 (35.7)	0.3
No	209 (76.8)	325 (74.4)	0.5	186 (68.4)	286 (65.6)	0.4	93 (34.2)	89 (20.5)	<0.0001	74 (27.4)	150 (35.0)	0.0371	64 (23.5)	100 (22.9)	0.8

### Factors associated with violence (Multivariate regression results: 4 different models)

On controlling for repeating a grade at school and sexual partnerships, Black (OR: 4.6, 95% CI: 2.7-7.9), Coloured (OR: 3.9, 95% CI: 2.0-7.4) and White (OR: 8.0, 95% CI: 4.0-16.2) adolescents were associated with a higher odds of witnessing acts of violence in the community (Table 3). Males (OR: 1.6, 95% CI: 1.1-2.2), Whites (OR: 2.1, 95% CI: 1.05-4.2), and ever taking alcohol (OR: 2.6, 95% CI: 1.7-4.0) were associated with higher odds of experiencing acts of violence in the community after controlling for repeating a grade at school. Females (OR: 1.8, 95% CI: 1.3-2.6), Blacks (OR: 2.2, 95% CI: 1.1-4.1), Whites (OR: 3.0, 95% CI: 1.4-6.4) and ever taking alcohol (OR: 2.9, 95% CI: 2.0-4.3) were associated with higher odds of witnessing violence in the family after controlling for repeating a grade at school. A higher odds of experiencing violence in the family was likely in Blacks (OR: 2.1, 95% CI: 1.1-3.9), single parent families (OR: 1.5, 95% CI: 1.01-2.1) and among those ever taking alcohol (OR: 1.8, 95% CI: 1.2-2.6).

**Table 3:** **Factors associated with violence**

	**Ever seen an act of violence in the community**		**Ever experienced an act of violence in the community**		**Ever seen violence in the family**		**Ever experienced violence in the family**	
	**Multivariate**		**Multivariate**		**Multivariate**		**Multivariate**	
	**OR (CI)**	**p-value**	**OR (CI)**	**p-value**	**OR (CI)**	**p-value**	**OR (CI)**	**p-value**
**Gender**								
Male	-	-	1.6 (1.1-2.2)	0.0143	Ref		-	-
Female	-	-	Ref		1.8 (1.3-2.6)	0.0008	-	-
**Ethnic group**								
Black	4.6 (2.7-7.9)	<0.0001	01.0 (0.5-1.7)	0.8	2.2 (1.1-4.1)	0.0183	2.1 (1.1-3.9)	0.0247
Coloured	3.9 (2.0-7.4)	<0.0001	0.9 (0.4-1.9)	0.8	2.0 (0.9-4.3)	0.1	1.4 (0.6-3.0)	0.4
Indian	Ref		Ref		Ref		Ref	
White	8.0 (4.0-16.2)	<0.0001	2.1 (1.05-4.2)	0.0367	3.0 (1.4-6.3)	0.0042	2.0 (0.9-4.2)	0.1
**Repeated Grade**								
Never repeated	Ref		Ref		Ref		Ref	
Repeated	1.5 (1.01-2.1)	0.0462	2.3 (1.6-3.3)	<0.0001	1.6 (1.1-2.2)	0.0117	1.4 (1.0-2.0)	0.1
**Parents Alive**								
Both parents alive	-	-	-	-	-	-	Ref	
Single parent alive	-	-	-	-	-	-	1.5 (1.01-2.1)	0.0423
None alive*	-	-	-	-	-	-	1.8 (0.9-3.6)	0.1
**Number of sexual partners**								
None or one	Ref		-	-	-	-	-	-
More than one	1.7 (1.1-2.5)	0.0115	-	-	-	-	-	-
**Ever had alcohol**								
Yes	2.1 (1.5-2.9)	<0.0001	2.6 (1.7-4.0)	<0.0001	2.9 (2.0-4.3)	<0.0001	1.8 (1.2-2.6)	0.0033
No	Ref		Ref		Ref		Ref	

NB: *Orphan.

## Discussion

Sadly exposure to community and family violence is endemic among adolescents from low socio-economic settings in all ethnic groups in Johannesburg, South Africa. Female adolescents are most affected by violence and are more likely to witness and experience violence within the family. Males are more likely to witness and experience violence in the community. Most adolescents appear to be witnessing violence rather than personally experiencing it. To better understand the burden of violence in South Africa, we purposefully included a broader base of ethnic groups compared to prior research. Although not necessarily ethnically representative, our findings were consistent with previous research [6,9,36].

Sexual violence is rife in low socio-economic settings in South Africa, and females bear the brunt with a high prevalence of sexual abuse and unwanted sexual advances noted amongst White adolescents. The effects of sexual abuse are reflected in higher sexual risk and use of substances [2,37]. As a result, adolescent sexual violence, both as perpetrators and victims is an important public health concern with implications on sexual and reproductive health. Our findings concur with previously reported work from South Africa [4,37]. The proportion of White adolescents reporting sexual violence was highest in our sample and requires further investigation.

Black, White and Coloured adolescents experienced more violence than Indian adolescents. Though the mechanisms of protection/risk for this disparity were not explored in our study, possible contributing reasons include poverty and substance use [5,38]. Alcohol use in our study is consistently associated with witnessing or experiencing acts of violence both in the family and community. However, Indian adolescents were less likely to have ever taken alcohol. The majority of adolescents who reported experiencing unwanted sexual advances were female and had consumed alcohol within the past six months. It may be that they explored use of alcohol as a coping mechanism for trauma [39]. Amongst adolescents, alcohol consumption is increasing globally and the debut of alcohol use is occurring at a younger age [23]. Our findings suggest that alcohol use may need to be addressed in any interventions on violence targeting adolescents in low-socioeconomic communities in Johannesburg.

A possible limitation of our study is recall bias. Our sample of adolescents, between the ages of 16 and 18 years, recalled their first violent experiences at the median age of 14 years. Violent experiences may have been under-reported, in general. Sexual abuse is likely under-reported [6,11], particularly in male adolescents probably due to stigma and fear of seeming ‘un-manly’ [40]. Measures were self-reported and possibly influenced by social desirability bias. The marked lower exposure to violence amongst the Indian population could be a true reflection or an underestimation as a result of social desirability bias or cultural inclinations toward privacy and/or secrecy [41]. In our study, causality cannot be inferred as this was a cross-sectional non-random sample. Though not generalizable, our findings may have relevance to other similar settings. We acknowledge the conflation between ethnicity and community in which confounding is introduced.

Addressing factors outside of the family could be more beneficial for males, whereas addressing factors within the family/household may provide better protection for females. Prior research suggests that exposure to family violence may affect development differently than exposure to community violence [42], which further supports our recommendation of identifying separate prevention methods for violence exposure that occurs within the family and that occurs within the community.

## Conclusions

Given the public health challenges attributed to violence, the high proportion of adolescents from low socio-economic settings exposed to violence in South Africa is a matter of concern. Interventions to mitigate the effects of violence are urgently required.
